# Puzzling Over the Pneumococcal Pangenome

**DOI:** 10.3389/fmicb.2018.02580

**Published:** 2018-10-30

**Authors:** N. Luisa Hiller, Raquel Sá-Leão

**Affiliations:** ^1^Department of Biological Sciences, Carnegie Mellon University, Pittsburgh, PA, United States; ^2^Center of Excellence in Biofilm Research, Allegheny Health Network, Pittsburgh, PA, United States; ^3^Laboratory of Molecular Microbiology of Human Pathogens, Instituto de Tecnologia Química e Biológica António Xavier, Universidade Nova de Lisboa (ITQB NOVA), Oeiras, Portugal; ^4^Departamento de Biologia Vegetal, Faculdade de Ciências, Universidade de Lisboa, Lisbon, Portugal

**Keywords:** *Streptococcus pneumoniae* (pneumococcus), pangenome, genomic diversity, genomic plasticity, horizontal gene transfer, competence, vaccine, antibiotics

## Abstract

The Gram positive bacterium *Streptococcus pneumoniae* (pneumococcus) is a major human pathogen. It is a common colonizer of the human host, and in the nasopharynx, sinus, and middle ear it survives as a biofilm. This mode of growth is optimal for multi-strain colonization and genetic exchange. Over the last decades, the far-reaching use of antibiotics and the widespread implementation of pneumococcal multivalent conjugate vaccines have posed considerable selective pressure on pneumococci. This scenario provides an exceptional opportunity to study the evolution of the pangenome of a clinically important bacterium, and has the potential to serve as a case study for other species. The goal of this review is to highlight key findings in the studies of pneumococcal genomic diversity and plasticity.

## Pneumococcal Vaccines and Antibiotics

Despite current vaccines and antibiotics, *Streptococcus pneumoniae* remains a leading cause of morbidity and mortality worldwide (O’Brien et al., 2009; Drijkoningen and Rohde, 2014). Pneumococcal infection can take several forms such as acute otitis media, pneumonia, bacteremia and meningitis (Pneumococcal Disease | Clinical | Streptococcus pneumoniae | CDC, 2017). Pneumococcal infection is preceded by colonization of the upper respiratory tract (mainly the nasopharynx), which is frequent and asymptomatic (Simell et al., 2012). As a colonizer, pneumococci live as a biofilm, an ideal environment for strain co-existence and horizontal gene transfer (Hall-Stoodley et al., 2006; Oggioni et al., 2006; Sanderson et al., 2006; Hoa et al., 2009; Marks et al., 2012a; Blanchette-Cain et al., 2013).

Most pneumococci have a polysaccharide capsule, an anti-phagocytic structure that surrounds the cell. There are nearly one hundred diverse capsular types (Bentley et al., 2006; Geno et al., 2015). Current prevention strategies of pneumococcal infection include the use of multivalent pneumococcal conjugate vaccines which target a subset of all capsular types selected based on their association to the most common and/or virulent isolates in circulation. The first multivalent conjugate vaccine targeted seven capsular types (PCV7) and was widely implemented in the United States in 2000, and subsequently in many countries across the globe. Currently, there are two vaccines (PCV10 and PCV13) being used worldwide. For a detailed review see Geno et al. (2015). These vaccines are effective in preventing disease and decreasing colonization due to vaccine serotypes (Pneumococcal Disease | Clinical | Streptococcus pneumoniae | CDC, 2017). Due to their limited valency, however, serotype replacement can occur (Weinberger et al., 2011). Disease replacement has been reported with different magnitudes depending on local epidemiology. Colonization replacement is extensive with no overall net decrease in prevalence (Lee et al., 2014; Nunes et al., 2016). As such, although the overall benefit of limited-valency pneumococcal conjugate vaccines is unquestionable, its benefits are expected to be eroded over time (Weinberger et al., 2011).

Management of pneumococcal disease includes the use of antibiotics. Although isolates of pneumococci in the pre-antibiotic era were susceptible to many antimicrobial agents, resistant isolates have been described since their introduction in clinical use (Frisch et al., 1943; Hamburger et al., 1943). In the 1960s, resistance to tetracycline (Evans and Hansman, 1963), macrolides (Dixon, 1967; Kislak, 1967), and penicillin was reported (Hansman and Bullun, 1967). In the 1970s, multidrug resistant strains, i.e., strains resistant to three or more classes of antibiotics, were described (Jacobs et al., 1978). By the late 1980s – early 1990s penicillin-resistant pneumococci, often multidrug-resistant – had spread globally, achieving extremely high incidence in some countries both as colonizing and disease-causing agents (Sá-Leão et al., 2000; McGee et al., 2001).

Ever since its first description in 1881, *S. pneumoniae* has been extensively studied leading to seminal scientific discoveries such as the putative use of polysaccharide antigens as vaccines (Avery et al., 1917), the ability of polysaccharides to induce antibodies (Heidelberger and Avery, 1923), bacterial gene transfer (Griffith, 1928), the first bacterial autolytic enzyme (Dubos, 1937), the isolation and chemical characterization of the first polysaccharide antigen (Goebel and Adams, 1943), the identification of the “transforming principle” (later named DNA) as the genetic material (Avery et al., 1944), the therapeutic efficacy of penicillin (Tillet et al., 1944), the role of bacterial capsule in resistance to phagocytosis (Felton et al., 1955), and the first bacterial quorum sensing factor (Tomasz, 1965).

In the genomic era, pneumococci continue to be intensively investigated. The first pneumococcal genome was published in 2001 (Tettelin et al., 2001). Currently, the genomes of over 8,000 strains are publicly available with a constant increase^1^. This scenario provides an exceptional opportunity to study the evolution of the pneumococcal pangenome under multiple selective pressures.

## The Pneumococcal Pangenome

**FIGURE 1:**
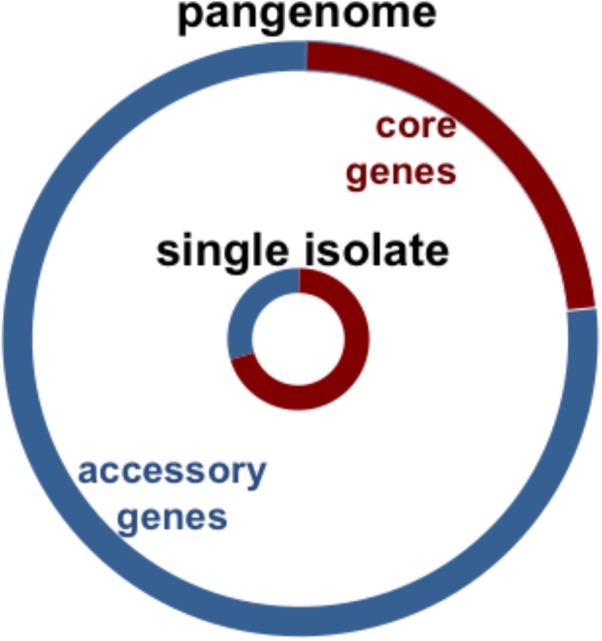
Chart illustrates pneumococcal genomic diversity. Distribution of orthologous clusters in a single isolate (inner circle), and the pangenome (outer circle). The core orthologous clusters are shared across all pneumococcal strains (red), and the accessory orthologous clusters are those not encoded in all strains, but instead unevenly distributed across isolates of this species (blue). The pangenome is the comprehensive set of all the orthologous clusters in the species.

The genes of the pneumococcus expand beyond those encoded within a single strain. Instead, they are distributed over the pneumococcal population, providing this species with an expanded set of genes to draw from for its own adaptation and evolutionary success (Hiller et al., 2007; Donati et al., 2010). As illustrated by the sequencing of the TIGR4 strain in 2001, pneumococcal genomes are approximately 2 megabases in length and encode an estimated 2200 coding sequences (Hoskins et al., 2001; Tettelin et al., 2001; Lanie et al., 2007; Ding et al., 2009). Over twenty percent of the coding sequences of any single pneumococcal isolate are not encoded in all strains, but instead are part of an accessory genome unevenly distributed across isolates of this species (Figure 1; Hiller et al., 2007; Donati et al., 2010). The pneumococcal core genome is estimated to encode 500–1100 clusters of orthologues (Donati et al., 2010; Croucher et al., 2013a; Gladstone et al., 2015; Tonder et al., 2017). In contrast, the pneumococcal pangenome is estimated to encode 5000–7000 clusters of orthologues (see Table 1 for definitions) (Donati et al., 2010; Croucher et al., 2013a; Gladstone et al., 2015; Tonder et al., 2017). In this manner, about three quarters of pneumococcal genes are differentially distributed across strains (Figure 1). Notably, in a study of isolates from the Maela refugee camp in Thailand, the pangenome structure was distinct from other studies: the pangenome was estimated at approximately 13,000 orthologous clusters and the core at approximately 400 (Tonder et al., 2017).

**Table 1:** Definition of terms used in this review.

Term	Definition	Comment
*Core Genome*	The set of clusters of orthologous genes shared by all strains in a species.	
*Accessory Genome*	The set of clusters of orthologous genes not shared by all strains in a species. Also known as distributed genome or dispensable genome.	These clusters of orthologues may be widely distributed across strains or unique to a single strain.
*Pangenome*	Comprehensive set of clusters of orthologous genes in a species. Also known as the Supragenome.	Sum of the core and the accessory genomes.
*Serotype replacement*	The increase in non-vaccine serotypes observed after implementation of the pneumococcal vaccine.	
*Serotype switching*	The process of changing the genes encoded in the capsular loci by recombination.	Serotype switches from a vaccine-type capsule to a non-vaccine type capsule is an important mechanisms of vaccine escape.
PubMLST and BiGSdb	The PubMLST database contains the whole genome sequences of thousands of pneumococcal genomes. The BIGSdb software on this database allows for genome-wide analyses.	
Global Pneumococcal Sequencing project (GPS)	Global initiative that uses whole genome sequencing to investigate the impact of pneumococcal vaccines on the pangenome.	

The accessory genome, with its diversity of genes and alleles, is a collection of genetic material. Thus, in a multi-strain biofilm, pneumococcal isolates can exchange DNA, generating strains with novel gene combinations, which may be beneficial to outcompete colonizers, evade host immunity, and escape human interventions, such as vaccines and antibiotics (Ehrlich, 2001; Ehrlich et al., 2005).

**FIGURE 2:**
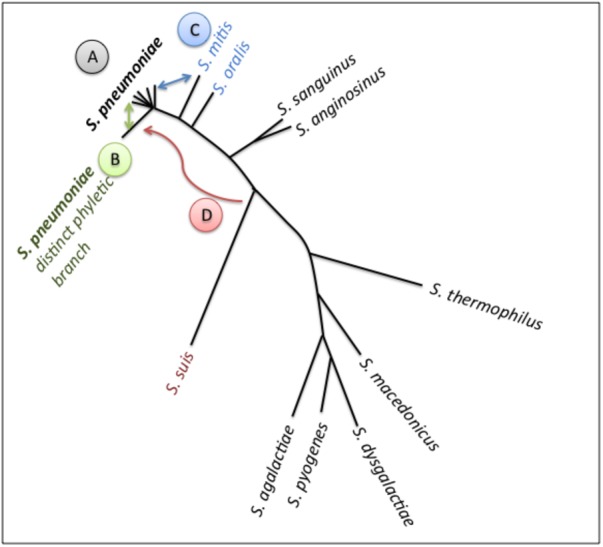
Schematic of pneumococcal genomic plasticity. Schematic is build on a phylogenetic tree of a subset of streptococcal species (adapted from Antic et al., 2017). Arrows indicate gene transfer events discussed in this article. **(A)** Pneumococcal genomes, shaped by recombination across strains. The rates of gene exchange vary across lineages. Non-encapsulated strains display high rates of transfer, and may serve as recombination highways. The PMEN1 lineage (type 81) has impacted the genomes of multiple lineages as a common gene donor. The PMEN31 lineage (serotype 3 lineage from clonal complex 180) has relatively stable genomes. **(B)** A distinct phyletic branch captures strain differentiation. It is composed of the classical non-typable strains, which colonize the nasopharynx and can cause conjunctivitis. Gene exchange between this branch and all other pneumococci may prevent speciation. **(C)** Gene transfer between *S. pneumoniae* and *S. mitis* has played a critical role in penicillin resistance via formation of mosaic PBPs. **(D)** Gene transfer between *S. suis* and *S. pneumoniae*, illustrated by the acquisition of an adhesin by pneumococci.

The origin of the accessory genome extends beyond this species (Figure 2). *S. mitis* also colonizes the human nasopharynx, and is the main reservoir of pneumococcal genetic diversity outside the species. Further, other colonizers of the upper respiratory tract, such as *S. pseudopneumoniae, S. oralis* and *S. infantis* are substantial contributors to the pneumococcal pangenome (Kilian et al., 2008; Donati et al., 2010). Finally, mobile elements from distantly related streptococci and other genera are additional sources of pneumococcal diversity (Courvalin and Carlier, 1986).

## Mechanisms of Pneumococcal Plasticity

The pneumococcus has evolved a striking propensity for genetic exchange. When combined with the extensive variability in pneumococcal genome content, these high rates of recombination can generate novel gene combinations within short time frames, such as during a single chronic infection (Hiller et al., 2010; Golubchik et al., 2012). Pneumococci spread from person-to-person via secretions from the upper respiratory pathway (Weiser et al., 2018). Thus, gene transfers that are incurred in the nasopharynx are the most likely to be transmitted, and the main source of long-term variation in the species. In general, bacteria employ transformation, conjugation and transduction for horizontal gene transfer; the pneumococcus makes use of all these mechanisms.

The pneumococcus is a classic example of natural competence. Via quorum sensing, bacterial cells produce and sense a peptide pheromone (competence stimulating peptide) that induces a state of competence (Tomasz and Hotchkiss, 1964; Havarstein et al., 1995). Competence leads to the regulation of over one hundred genes, including the apparatus for DNA uptake and transformation (Dagkessamanskaia et al., 2004; Peterson et al., 2004). While transformation frequencies vary across strains and conditions, transformation in pneumococcus can be highly efficient. In biofilm grown cells in a bioreactor, transformation reaches rates on the order of 10^-3^ (Lattar et al., 2018). It is noteworthy, that in these conditions recombination between strains may be unidirectional, although the mechanism behind this intriguing observation remains to be uncovered (Lattar et al., 2018). In animal models of dual-strain carriage, transformation reaches rates on the order of 10^-2^ (Marks et al., 2012b). The representative length of a recombinant fragment resembles that of a gene, at approximately 1 Kb (Donati et al., 2010). However, under selective pressure and in environments that involve cell-to-cell contact (such as an *in vitro* biofilm or the nasopharynx) much larger recombinant fragments are observed, often with lengths substantially over 10 Kb (Trzciński et al., 2004; Brueggemann et al., 2007; Hiller et al., 2010; Golubchik et al., 2012; Cowley et al., 2018).

Multiple factors contribute to pneumococcal transformation. First, pneumococcal biofilms provide abundant DNA. The biofilm matrix is DNA-rich and pneumococci encode competence-induced bactericidal molecules that lyse neighboring cells exposing DNA from divergent strains (Håvarstein et al., 2006; Dawid et al., 2007; Hall-Stoodley et al., 2008). Second, pneumococci can protect internalized ssDNA. Cells produce high amounts of single stranded DNA binding (SsbB) protein, which protects up to half a genome equivalent of intracellular DNA. These ssDNA-SsbB complexes serves as DNA reservoirs that can be used in multiple recombination events (Attaiech et al., 2011). Third, the recombination machinery does not require long stretches of identical sequence for recombination, this allows for the incorporation of highly divergent alleles or novel genes (Prudhomme et al., 2002). Moreover, human interventions can also influence transformation. Specifically, multiple classes of antibiotics can activate competence (Prudhomme et al., 2006; Stevens et al., 2011; Slager et al., 2014; Domenech et al., 2018). Three independent mechanisms have been described. First, aminoglycosides trigger an increase in misfolded proteins, which are substrates for the HtrA protease that normally degrades CSP. This substrate competition increases the pool of available CSP (Stevens et al., 2011). Second, fluoroquinolones and HPUra stall replication, and in doing so increase the dosage of the competence genes that are located near the origin of replication (Slager et al., 2014). Third, aztreonam and clavulanic acid drive cell chaining, increasing local concentrations of CSP and promoting competence (Domenech et al., 2018). In conclusion, the pneumococcus has evolved multiple strategies to ensure high rates of transformation and human intervention can further increase these rates.

Transduction is the horizontal transfer of genes via bacteriophages. These phages display lysogenic and lytic cycles. In the lysogenic phase, prophages are integrated into the host genome and vertically transmitted. In the lytic phase, phage excise from the host genome, undergo replication, lyse the host cell and are horizontally transmitted through infection of other bacterial cells. Prophages are ubiquitous within pneumococcal genomes, and there is clear evidence of phage activity in the species (Mcdonnell et al., 1975; Bernheimer, 1977; Ramirez et al., 1999; Romero et al., 2009a,b; Brueggemann et al., 2017). The majority of pneumococcal prophages can be organized into five groups based on sequence similarity. Furthermore, the phage integrase defines the genomic location where a phage will integrate into the genome, and most pneumococcal prophages are localized in one of five conserved locations (Brueggemann et al., 2017). Pneumococcal prophages display a highly variable strain distribution, consistent with extensive gene transfer, gain and loss, as well as restriction modification systems that limit acquisition of certain phages in subsets of strains (Croucher et al., 2014b). Nonetheless, in the mist of such plasticity, there are associations between specific prophages and pneumococcal lineages as exemplified by the ϕMM1 phage. This phage is integrated in strains of the PMEN1 lineage, a well-studied pandemic multidrug penicillin-resistant lineage also referred to as the Spain 23 F (ST81) clone, where it has persisted for decades (Muñoz et al., 1991; Corso et al., 1998; Ramirez et al., 1999; Sá-Leão et al., 2000; McGee et al., 2001; Obregón et al., 2003; Brueggemann et al., 2017). Studies on a ϕMM1-like phage have shed light on the potential role of phages in pneumococcal biology: ϕMM1-like phages promote adherence to inert surfaces and pharyngeal cells in multiple genomic backgrounds (Loeffler and Fischetti, 2006). The link between phage proteins and adherence is not specific to this group of phages, the phage tail protein PblB (not encoded by the MM1 group) also promotes adherence to human epithelial cells, as well as nasopharyngeal and lung infection in a murine model of pneumococcal disease (Hsieh et al., 2015). Finally, the Spn1 prophage offers an example of the impact of a phage directly on pneumococcal physiology. Spn1 causes a defect in LytA-mediated autolysis and an increase in chain length, it decreases fitness in the mouse model of asymptomatic colonization, and it affords increased resistance to lysis by penicillin (DeBardeleben et al., 2014). Together these findings are consistent with the hypothesis that at least some groups of prophages modify the fitness of their pneumococcal hosts and provide an evolutionary framework to study the long-term persistence of subsets of phages within this bacterial species. The vast majority of phage-encoded coding sequences have unknown functions, and their role in pneumococcal biology and evolution remains an exciting open question.

Pneumococcal genomes also encode phage-related chromosomal islands (PRCIs) (Croucher et al., 2014b; Javan and Brueggemann, 2018). Unlike prophages the PRCIs are not capable of lytic cycles, instead they are presumed to hitchhike with prophages. PRCIs encode genes that enable use of the phage-reproduction machinery of other phages; they can be conceptualized as phage parasites (Novick et al., 2010). The PRCI distribution across lineages appears much less diverse than that of prophages, perhaps due to limited lateral transfer or due to high specificity between PRCI and subsets of prophage hosts. The pneumococcal PRCIs can be organized into four major groups (Javan and Brueggemann, 2018). The molecular machinery of pneumococcal PRCIs, and their role in genomic plasticity and pneumococcal biology is largely unknown.

Conjugation is employed by pneumococci for horizontal gene transfer of integrative and conjugative elements (ICEs), which consist of conjugative transposon or integrative plasmids. Conjugation requires direct contact between donor and recipient cells. When inserted into the bacterial genome these autonomous mobile elements undergo vertical transfer. Alternatively, they can be excised from the genome, undergo horizontal gene transfer across strains, and integrate into the recipient’s genome (Burrus et al., 2002). Many of the best-characterized pneumococcal ICEs are related to Tn5253, and correspond to composite transposons. These elements are found in variable locations within the bacterial genome, consistent with independent insertion events (Ayoubi et al., 1991; Croucher et al., 2009; Chancey et al., 2015). ICEs are particularly important to pneumococcal biology given their propensity to carry drug resistant genes, including resistance to macrolides, tetracycline, and chloramphenicol (Ayoubi et al., 1991; Croucher et al., 2009; Chancey et al., 2015). ICEs can carry multiple kilobases of genomic material, and as such allow for substantial genomic diversity to emerge from a single transfer event. A well-studied instance of an ICE that carries multiple drug resistance determinants is ICESp23FST81, a Tn5353 found within many strains of the PMEN1 lineage. This element encodes multiple drug resistant genes, and has gained additional ones via integration of the Tn916 element into the original Tn5252 ICE. Further, it also encodes the TprA2/PhrA2 quorum sensing signaling system, which signals to itself as well as to a homologous quorum sensing system widely distributed in the species (TprA/PhrA). Downstream and negatively regulated by TprA2, is a lanthipeptide cluster that promotes disease in a murine model of pneumonia (Kadam et al., 2017). While ICESp23FST81 is almost exclusive to PMEN1, it is also found outside this lineage in a ST156 strain depicting an instance of ICE transfer across diverse lineages (Chancey et al., 2015). ICEs and PRCIs display mosaic patterns that are consistent with variation via transformation events. Thus, these mobile elements reveal how transduction or conjugation cooperate with transformation to generate pneumococcal diversity (Croucher et al., 2014b; Chancey et al., 2015).

In summary, transformation, transduction and conjugation drive the evolution of pneumococcus. Recombination plays a pivotal role in generating vaccine escape strains via capsular switching and drug resistant strains via the acquisition of resistance alleles or genes.

## Gene Transfer in the Pneumococcus: Implications for the Pangenome and Pneumococcal Biology

Pneumococcus researchers have the privilege of studying a species with an abundance of genomic data (Jolley and Maiden, 2010; Riley et al., 2012). There are multiple genome-wide studies, including detailed annotations of single genomes and comparative analyses of lineages, serotypes, and populations (Dopazo et al., 2001; Hoskins et al., 2001; Tettelin et al., 2001; Hiller et al., 2007; Lanie et al., 2007; Croucher et al., 2009, 2011, 2013a, 2014b; Ding et al., 2009; Donati et al., 2010; Everett et al., 2012; Wyres et al., 2012; Chewapreecha et al., 2014; Chaguza et al., 2016b, 2017; Lees et al., 2017; Makarewicz et al., 2017; Aprianto et al., 2018; Azarian et al., 2018). The selective pressure imposed by multivalent pneumococcal conjugate vaccines, provides a unique opportunity to observe population dynamics and investigate the pangenome under an instance of dramatic selection. The GPS (**G**lobal **P**neumococcal **S**equencing **p**roject) is an ongoing remarkable global initiative amidst several others aiming to employ genome wide studies to understand the impact of the vaccine on the pangenome^2^.

The origin of non-vaccine type strains post-vaccination is of great importance for vaccine implementation and public health. In these circumstances serotype replacement occurs mainly due to expansion of non-vaccine types that pre-exist before vaccine introduction (Simões et al., 2011; Frazão et al., 2013; Chaguza et al., 2017). A study in Portugal, observed that the overall prevalence of drug-resistant pneumococci among carriers, commonly associated with vaccine serotypes in the pre-vaccine era, did not change following PCV7 use despite extensive serotype replacement (Simões et al., 2011). This was due to expansion of drug-resistant lineages expressing non-vaccine types that were already in circulation, albeit at lower frequency, in the pre-vaccine era. Perhaps contrary to expectations, post-vaccine capsular switches of vaccine types to non-vaccine types did not contribute significantly to the pool of post-vaccine drug resistant isolates. Nonetheless, there is at least one example of serotype switch leading to maintenance, in the post-vaccine era, of a clone previously associated with a vaccine type. The PMEN14 lineage, also referred to as Taiwan 19F, was a highly prevalent lineage and a major contributor to pneumococcal disease in the PCV7 era. While the 19F capsule was targeted by PCV7, its serotype switch to serotype 19A was not and spread in the United States in the PCV7 era (Geno et al., 2015). A large comparative analysis of PMEN14 isolates detected multiple instances of serotype switching to 19A (Croucher et al., 2014a). Notably, a study of strains from non-vaccinated volunteers also captured multiple instances of gene transfer into the PMEN14 lineage (Chewapreecha et al., 2014; Croucher et al., 2014a). These studies support the model where gene exchange within a lineage generates a mixed population, which is situated to withstand selective pressures such as those imposed by the vaccines (Croucher et al., 2014a). Once selective pressure is imposed, it results in the spread of the genotypes with a fitness advantage; the subsequent competition across these genotypes, shapes the population.

Akin to fitness, linkage disequilibrium is another player in the outcome of recombination in pneumococci. A comparative genomic analysis combined with mathematical models suggests that pneumococci display clear groups of alleles, termed metabolic types (Watkins et al., 2015). This analysis categorized allelic differences in 876 metabolic/transport genes; it demonstrated a highly non-random distribution of genes and higher linkage disequilibrium within this set when compared to control sets of genes. These metabolic types represent a fitness peak, which may be decreased by recombination events. In a more recent work, it is proposed that epistatic interaction between the groEL chaperone and other genes may be a strong driving force in this process (Lourenço et al., 2017). Moreover, metabolic types are associated with serotypes and thus are likely to influence the fitness of serotype switches. It seems probable that the 19F–19A switches described above do not disrupt metabolic profiles. The authors propose that genes within a metabolic type have co-evolved, and represent a highly successful set of alleles that are well adapted to a particular metabolic niche. In conclusion, gene exchange plays a critical role in the evolution of pneumococci, but may be limited by decreased fitness of recombinant strains and linkage disequilibrium.

### Evidence of *in vivo* Recombination: Characterization, Tempo, and Barriers

The first documented example of *in vitro* recombination is the Griffith (1928) experiment. A non-encapsulated strain incorporated DNA from an encapsulated isolate, leading to its conversion from avirulent to virulent (Griffith, 1928). Building on this eminent experiment, the pneumococcal community has captured and quantified *in vivo* recombination.

Pneumococcal gene transfer is common across strains in the same lineage. This can be exemplified by exchange across strains of the pandemic PMEN1 lineage. A study comparing 240 PMEN1 isolates, recovered across the globe over 25 years, captured over 700 recombination events (Croucher et al., 2011). These events were concentrated in the capsular locus, regions encoding surface exposed molecules implicated in host interactions (*pspA, psrP*, and *pspC*), as well as the ICESpn23FST81 and the ϕMM1-2008 prophage. While recombination across PMEN1 isolates is widespread, these strains have a low propensity to receive heterologous DNA (Wyres et al., 2012). Ninety five percent of the coding sequences from modern PMEN1 strains are highly similar to those of their common ancestor, represented as an isolate from 1967. It has been proposed, that the DpnIII restriction modification system, which is encoded in the vast majority of PMEN1 strains and rare outside the lineage, provides one mechanism to decrease the frequency of heterologous recombination in this group and in doing so, fosters genomic stability in the PMEN1 lineage (Eutsey et al., 2015). In contrast, strains in the PMEN1 lineage display a high propensity to donate DNA and have substantially impacted the genomic composition of many lineages (Wyres et al., 2012). The genes that encode a subset of the penicillin binding proteins (PBPs) confer penicillin non-susceptibility; the alleles from the PMEN1 lineage are widely distributed across the pneumococcal population (Sá-Leão et al., 2002; Wyres et al., 2012). In addition, the PMEN3 and CGSP14 lineages, have acquired 5.3 and 9.5% of their genomes from the PMEN1 lineage, respectively (Wyres et al., 2012). Combined, these studies capture recombination *in vivo*, describe the uneven transfer of genes across lineages, and illustrate an example of extensive gene exchange within a single pneumococcal lineage.

New gene combinations can be generated in the time frame of a chronic mucosal infection or a colonization event. A study of a single, chronic and polyclonal infection captured the progressive accumulation of recombinations (Hiller et al., 2010). Specifically, it compared a set of six clinical strains, isolated over a 7-month period from a single child. This set contains the major parental strain, the DNA donor, and the recombinant and thus allowed the reconstitution of inter-lineage i*n vivo* recombination replacements. A substantial percentage of the genome, over seven percent of the dominant lineage, was recombined over the course of this single infection. The transferred regions were distributed over the genome consistent with 23 recombination events. A separate study captured multiple genes donated by a single donor in a population analysis of vaccine escape strains (Golubchik et al., 2012). It documented transfers, ranging from 0.04 to 44 Kb in size, in the capsular locus and across the genome. Together, these studies capture multiple instances of *in vivo* transfer between strains. Their findings are consistent with multiple transfers during a single competence event and with consecutive sequential events between donor and recipient strains during co-existence in a biofilm.

### Inter-Species Gene Transfer

A prominent example of inter-species gene exchange is documented in the evolution of penicillin resistance (Figure 2, gene exchange between C and A). The main genetic determinants of penicillin resistance are variable alleles of the penicillin-binding proteins PBP1A, PBP2B and PBP2X. Resistance occurs, when alleles of these cell wall synthesis proteins display decreased binding affinity to penicillin (Hakenbeck et al., 1980; Zighelboim and Tomasz, 1980; Grebe and Hakenbeck, 1996; Smith and Klugman, 1998). The genes coding for these low affinity PBPs are mosaic genes formed by recombination between strains, in many cases between pneumococcus and other streptococcal species (Dowson et al., 1989; Sibold et al., 1992; Chi et al., 2007; Sauerbier et al., 2012; Mousavi et al., 2017). A recent study captured a gene exchange event between a strain of *S. mitis* and pneumococcus isolated from a longitudinal study in a cystic fibrosis patient (Rieger et al., 2017). An additional example for a functional class of genes that undergoes exchange across species is peptides implicated in cell-cell communication. These coding sequences can display allelic distributions that do not match that of the species tree, suggestive of intra and inter-species gene exchange (Hoover et al., 2015; Cuevas et al., 2017; Kadam et al., 2017). Their presence and distributions outside pneumococcus are consistent with both gene exchange and coordinated interactions across species boundaries.

### Differences in Plasticity Across Lineages

Pneumococcal lineages differ in their plasticity. Non-encapsulated strains appear to have the highest recombination frequencies. A study of over 3,000 carriage genomes, from residents of the Maela refugee camp in Thailand, observed the highest frequency of gene donors and recipients in non-encapsulated strains (Chewapreecha et al., 2014). These findings are highly relevant to public health as they suggest that strains beyond the scope of the vaccines serve as highways of recombination. In contrast, the lineage encompassing the serotype 3 strains from clonal complex 180 stand out as a lineage with a very stable genome and rare transfer events (Croucher et al., 2013b). This example is particularly significant as serotype 3 is targeted by PCV13, yet serotype 3 isolates are prevalent in some populations post PCV13 (Horácio et al., 2016; Slotved et al., 2016; Lapidot et al., 2017; Silva-Costa et al., 2018). The explanation for the possible reduced efficacy of the vaccine toward serotype 3 strains may lie in the fact that, unlike most capsules, their mucosal capsule is not covalently linked to the cell wall (Caimano et al., 1998; Cartee et al., 2000). Studies in the murine model suggest that type 3 capsule can detach from the bacteria and serve as decoy diverting the antibody response away from the bacteria and in doing so decreasing vaccine efficacy (Choi et al., 2016). These type 3 strains are highly virulent and display a relatively low duration of carriage (Sleeman et al., 2006). Thus these extreme differences in plasticity may be attributed, at least in part, to differences in virulence potential and duration of carriage, which are associated with transformation efficiency (Chaguza et al., 2016a).

### Balancing Selection of Accessory Genes

What is the consequence of vaccination on the pangenome composition? Given that accessory genes are often associated with lineages, what happens to the distribution of accessory genes associated with lineages of vaccine serotypes? One expectation is that as lineages decrease in frequency due to vaccine pressure, so do the accessory genes associated with such lineages. However, recent theoretical work and population studies suggest an alternative, where the frequency of accessory genes within a population returns to a baseline frequency after disruption by the vaccine (Corander et al., 2017; Azarian et al., 2018).

The mechanism proposed for balancing accessory genes within the pneumococcal population is negative frequency-dependent selection (Corander et al., 2017). A comparison of multiple pneumococcal populations suggests that the frequency of many accessory genes in a pneumococcal population is maintained even after major disruptions in the primary lineages via vaccination (Corander et al., 2017). The same trend was observed in a large genomic study that compared over 900 genomes from two Native American communities (Azarian et al., 2018). Strains were collected over a period of 14 years from three groups: a vaccine naïve population, a population soon after introduction of the vaccine, and a population four to 6 years post-vaccine. Over time, accessory genes trended to frequencies observed in the naïve population (Azarian et al., 2018). Together, these studies provide building evidence that selection from the human immune system and/or other microbes serve to equilibrate the composition of the pneumococcal pangenome. When combined with the notion of metabolic types, this observation has profound implications regarding our ability to predict the influence of vaccines and therapies on the pangenome composition and consequently on our ability to design effective long-term tools for prevention, diagnosis, and treatment of pneumococcal disease.

### Strain Diversification: A Distinct Phyletic Group Among the Pneumococcus

Within the genetically diverse pneumococci there is a clear example of strain diversification. A subset of non-encapsulated strains has long been identified as distinct in clinical studies (Porat et al., 2006; Williamson et al., 2008; Zegans et al., 2009; Norcross et al., 2010; Haas et al., 2011). More recently, four comparative genomic studies have observed that a subset of the non-encapsulated strains is clustered into a distinct phyletic branch (Croucher et al., 2014b; Hilty et al., 2014; Valentino et al., 2014; Antic et al., 2017). These are referred to as the classical non-encapsulated strains (Keller et al., 2016). Not all non-encapsulated strains are part of this distinct phyletic branch. The sporadic non-encapsulated strains belong to the major pneumococcal phylogenetic branch. For more detail, a comprehensive review of non-encapsulated strains is available (Keller et al., 2016). In this manner, non-encapsulated pneumococci can be classified into two highly distinct phylogenetic groups based on their core genome sequence, where the classical set represents a distinct phyletic branch (Figure 2, branch “B”).

The classical non-encapsulated strains encode a distinct accessory genome (Croucher et al., 2014b; Hilty et al., 2014; Valentino et al., 2014; Antic et al., 2017). This discrete set of genes has biological implications, as the vast majority of isolates that cause pneumococcal conjunctivitis are part of this group (Hilty et al., 2014; Valentino et al., 2014). The adhesin SspB exemplifies a gene product that is widely distributed in classical non-encapsulated strains and absent outside this set. SspB enhances attachment to ocular epithelial cells and may play a role in tissue tropism by extending the pneumococcal niche to the conjunctiva (Antic et al., 2017). A theoretical study on speciation has explored this distinct pneumococcal lineage to model speciation. The study suggests that frequent recombination between the classical non-encapsulated strains and other pneumococci maintains this branch at a constant genetic distance and may serve as a force to prevent speciation (Marttinen and Hanage, 2017). In summary, the classical non-typable strains illustrate strain diversification in the pneumococcus, and serve as the most extreme example of genetic diversity within this species.

## Perspectives

The pneumococcal community is poised for a new set of challenges and discoveries, with genomic data, *in vitro* and *in vivo* evolution studies, and omics technologies developing at unprecedented speeds. The use of antibiotics and the implementation of pneumococcal vaccines have played a crucial role in decreasing mortality due to pneumococcal infections. However, pneumococcus remains a major human pathogen, with high rates of carriage, multi-drug resistance, and serotype replacement (Huang et al., 2009; O’Brien et al., 2009; Rodrigues et al., 2009; Lee et al., 2014; WHO | Pneumococcal Disease, 2014; Nunes et al., 2016). There is significant progress in the development of a universal serotype-independent vaccine. These efforts are focused on protective and widespread protein antigens as well as on the development of whole cells vaccines (Miyaji et al., 2013; Rosch, 2014; Moffitt and Malley, 2016; Wilson et al., 2017). In parallel, experimental evolution in *in vitro* biofilms provide insights into adaptation *in vivo* (Churton et al., 2016; Cowley et al., 2018). Current studies suggest the revolutionary idea that we may be in a position to predict the genomic composition of dynamic pneumococcal populations and their responses to vaccines and therapies. Moreover, in keeping with pneumococcus as a model for genomic studies, these findings may be applicable to the evolution of pangenomes in other bacterial species. When integrated with future advances in the understanding of virulence determinants and master regulators of pathogenesis, such information should permit exquisitely effective vaccines and therapy implementation. Perhaps, one day, we may have the required knowledge to manipulate bacterial pangenomes to transform opportunistic pathogens into human commensals.

## Author Contributions

NH and RS-L had written the review.

## Conflict of Interest Statement

The authors declare that the research was conducted in the absence of any commercial or financial relationships that could be construed as a potential conflict of interest. The reviewer JR declared a past co-authorship with one of the authors NH to the handling Editor.

## Footnotes

1 https://pubmlst.org/

2 https://www.pneumogen.net/gps/project_outline.html
